# Biostimulatory Effects of *Chlorella fusca* CHK0059 on Plant Growth and Fruit Quality of Strawberry

**DOI:** 10.3390/plants12244132

**Published:** 2023-12-11

**Authors:** Young-Nam Kim, Jun Hyeok Choi, Song Yeob Kim, Young-Eun Yoon, Hyeonji Choe, Keum-Ah Lee, Vimalraj Kantharaj, Min-Jeong Kim, Yong Bok Lee

**Affiliations:** 1 Division of Applied Life Science (BK21), Gyeongsang National University, Jinju 52828, Republic of Korea; 2 Institute of Agriculture and Life Science (IALS), Gyeongsang National University, Jinju 52828, Republic of Korea; 3 Organic Agriculture Division, National Academy of Agriculture Science, Rural Development Administration, Wanju 55365, Republic of Korea

**Keywords:** green algae, biofertilizer, crop performance, fruit flavor, postharvest quality

## Abstract

Green algae have been receiving widespread attention for their use as biofertilizers for agricultural production, but more studies are required to increase the efficiency of their use. This study aimed to investigate the effects of different levels of *Chlorella fusca* CHK0059 application on strawberry plant growth and fruit quality. A total of 800 strawberry seedlings were planted in a greenhouse and were grown for seven months under different *Chlorella* application rates: 0 (control), 0.1, 0.2, and 0.4% of the optimal cell density (OCD; 1.0 × 10^7^ cells mL^−1^). The *Chlorella* application was conducted weekly via an irrigation system, and the characteristics of fruit samples were monitored monthly over a period of five months. The growth (e.g., phenotype, dry weight, and nutrition) and physiological (e.g., Fv/Fm and chlorophylls) parameters of strawberry plants appeared to be enhanced by *Chlorella* application over time, an enhancement which became greater as the application rate increased. Likewise, the hardness and P content of strawberry fruits had a similar trend. Meanwhile, 0.2% OCD treatment induced the highest values of soluble solid content (9.3–12 °Brix) and sucrose content (2.06–2.97 g 100 g^−1^) in the fruits as well as fruit flavor quality indices (e.g., sugars:acids ratio and sweetness index) during the monitoring, whilst control treatment represented the lowest values. In addition, the highest anthocyanin content in fruits was observed in 0.4% OCD treatment, which induced the lowest incidence of grey mold disease (*Botrytis cinerea*) on postharvest fruits for 45 days. Moreover, a high correlation between plants’ nutrients and photosynthetic variables and fruits’ sucrose and anthocyanin contents was identified through the results of principal component analysis. Overall, *C. fusca* CHK0059 application was found to promote the overall growth and performance of strawberry plants, contributing to the improvement of strawberry quality and yield, especially in 0.2% OCD treatment.

## 1. Introduction

Over the past century, inorganic fertilizers have been indispensable for improving crop production and quality, thereby ensuring food security in the face of a rapidly growing global population [1]. In addition, the chemical fertilizers used in agricultural lands, along with pesticides and herbicides, are judged to have, to date, met the stability of global food production to some extent [2,3]. However, excessive use of these agrochemicals has caused severe adverse impacts on the natural environments: e.g., soil acidification, mineral depletion, disease incidence, heavy metal contamination, eutrophication, biodiversity loss, and global warming [1,4,5], thereby exacerbating soil quality and health and subsequently reducing crop yield. In turn, these harmful effects, caused by chemical abuse, raise public concerns about food safety and security worldwide [6]. Hence, eco-friendly approaches to the improvement of crop yield and quality as well as efficiency in land management should be explored for sustainable agriculture.

Biofertilizers are substances containing microorganisms (e.g., bacteria, fungi, protozoa, and algae) or organic additives containing natural compounds derived from the microbes that promote plant growth and development by increasing the supply of essential nutrients (e.g., N, P, K, etc.) to the host plants [7]. It has been estimated that the application of biofertilizers may reduce the use of N–P–K fertilizers by up to 60% but increase crop yield by 35% [8]. Additionally, recent studies have demonstrated the superior effects of biofertilizer application on promoting the plant’s resistance to pathogens and abiotic stresses [9,10] as well as improving soil structure and water retention capacity [11]. Moreover, the fact that it is eco-friendly and cost-effective is increasing the global interest in biofertilizer development and production, mainly in the USA, Europe, China, and India. Accordingly, as of 2022 the global biofertilizer market was valued at US$2.15 billion and is projected to grow to US$6.83 billion in 2032, at a compound annual growth rate of 12.3% from 2023 to 2032 [12]. 

The major biofertilizer types include symbiotic (e.g., *Rhizobium*) or non-symbiotic (e.g., *Azotobacter*) N fixers, P solubilizing bacteria (e.g., *Aspergillus*, *Penicillium*), mycorrhizae (e.g., *Glomus*, *Scutellospora*), plant growth promoting bacteria (PGPR; e.g., *Agrobacterium*, *Pseudomonas*), vermicompost, cyanobacteria, etc. [2,4,13,14]. Among these, rhizobia-based biofertilizers account for approximately 80% of the global demand, followed by phosphate-mobilizing biofertilizers (approximately 15%) [15,16]. Albeit less frequent in agricultural use than these microbes, algae containing amino acids, fibers, plant nutrients, vitamins, and antioxidants [7,17] have also been reported for their high potential as biofertilizers. According to the results of many studies, the application of algae extracts considerably increased the yield and quantity of various crops and fruits, including maize, tomato, lettuce, spinach, chives, grape, mango, etc. via foliar or soil applications [18,19,20,21,22]. In addition, it has been confirmed that the algae applications induce enhancement of plant tolerances to pathogen attacks and abiotic stresses (e.g., salinity, drought, etc.), contributing to the promotion of the plant’s survival and performance [23]. 

In South Korea, freshwater algae, i.e., *Chlorella* spp., have been attracting great attention for their application to some leafy vegetables and strawberries (*Fragaria* × *ananassa*) since the mid-2010s. According to a report by the Rural Development Administration (RDA) [24], as of 2020 *Chlorella* farming practices were being applied to 66 crops and to a cultivation area of approximately 3700 ha. Additionally, it was estimated that farm income has increased by about 20% through *Chlorella* application. Kim et al. [25] revealed that the application of the *Chlorella vulgaris* CHK0008 strain to two strawberry cultivars, such as ‘Seolhyang’ and ‘Yukbo’, via soil irrigation or foliar treatment led to an increase in soluble solid content and a decrease in the decay rate of strawberry fruits. In addition, the beneficial effects induced by *Chlorella fusca* CHK0059, such as promoting the growth of strawberry plants and suppressing the Fusarium wilt disease of the fruits were reported [26]. Likewise, the role of *Chlorella* spp. as a biofertilizer and biocontrol agent has also been demonstrated in other studies [27,28,29]. As such, and based on these positive effects, *Chlorella* fertilization is widely used in the strawberry industry in Korea, though there is a lack of mechanism studies on the effects of *Chlorella* application on strawberry growth and fruit quality through monitoring experiments. Here, we aimed to investigate the application effects of *Chlorella fusca* CHK0059 on the morphological and physiological traits of strawberry plants and the quality and antifungal ability of the fruits. Then, through a comprehensive result analysis, we attempted to identify key factors in the beneficial effects of *Chlorella* fertilization and reveal their interactions. 

## 2. Results

### 2.1. Plant Characteristics

#### 2.1.1. Plant Biomass and Nutrient Content

**Figure 1:**
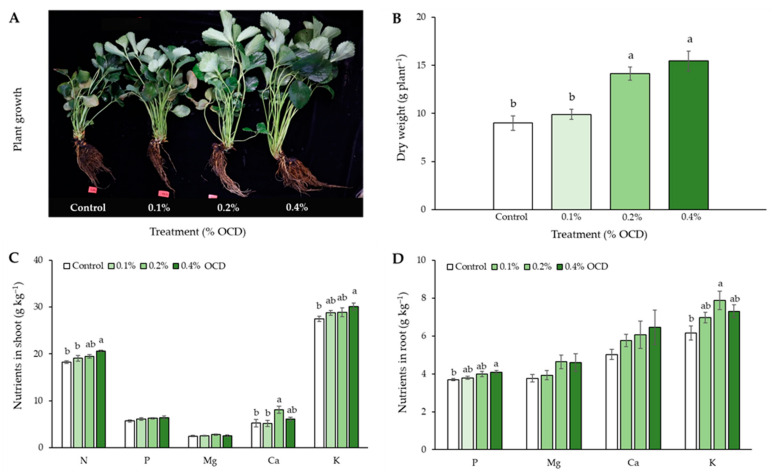
Phenotype (**A**), dry weight (**B**), and nutrient content in shoot (**C**) and root (**D**) of strawberry plants grown under different *Chlorella* application rates: 0, 0.1, 0.2, and 0.4% of the optimal cell density (OCD). Data are mean ± standard errors (*n* = 6). Different letters indicate significant difference among the treatments (Tukey’s HSD, *p* < 0.05).

The growth of strawberry plants varied greatly with *Chlorella* application rates, as shown in Figure 1A. The stem length and leaf size appeared to be greater as the application rate increased. Consistent with this trend, the highest dry weight (DW) of strawberry plants was observed in 0.4% of the optimal cell density (OCD; 1.0 × 10^7^ cells mL^−1^) treatment (15.5 g plant^−1^; Figure 1B). The DW values were significantly greater in 0.2% and 0.4% OCD treatments (1.57 and 1.72 times, respectively) than control (*p* < 0.05).

It was found that the higher the *Chlorella* application rate, the higher the nutritional level of strawberry plants. Among the nutrients, N, P, and K in the plant shoots were highest at 0.4% OCD (21, 6.4, and 30 g kg^−1^, respectively), while 0.2% OCD represented the highest concentrations of Ca (8.1 g kg^−1^), significantly higher than those in control (*p* < 0.05; Figure 1C). Additionally, the highest values of nutrients in the plant roots were observed in 0.2% and 0.4% OCD treatments for K and P, respectively (Figure 1D). 

#### 2.1.2. Photosynthetic Rate and Pigments

Table 1 shows the difference in the photosynthetic properties of plant leaves according to the *Chlorella* application rates. The maximum photosystem II quantum yield (Fv/Fm) was significantly higher in 0.2% and 0.4% OCD than in control (*p* < 0.05). In addition, chlorophyll (Chl) parameters, such as Chl a, Chl b, and total Chl, showed significantly higher values in 0.4% OCD than in control (*p* < 0.05). The carotenoid contents in the three OCD treatments (2.26–2.41 mg 100 g^−1^) were significantly higher than that in control (1.92 mg 100 g^−1^) (*p* < 0.05). 

### 2.2. Fruit Characteristics during the Monitoring Period

#### 2.2.1. Hardness, Soluble Solids Content, and Biomass

**Figure 2:**
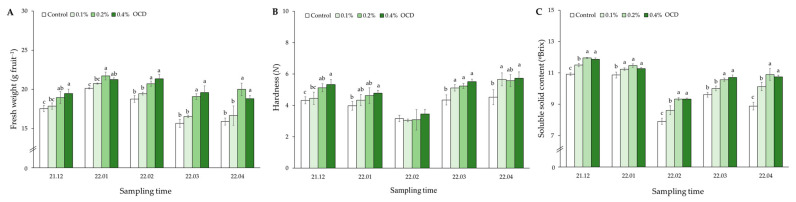
The properties of strawberry fruits grown under different *Chlorella* application rates for five months: 0 (control), 0.1, 0.2, and 0.4% of the optimal cell density (OCD). Data are mean ± standard errors (*n* = 50 for fresh weight and 6 for hardness and soluble solid content). Different letters indicate significant difference among the treatments (Tukey’s HSD, *p* < 0.05). (**A**) Fresh weight, (**B**) hardness, (**C**) soluble solid content.

The properties of strawberry fruits monitored during the five months (from December 2021 to April 2022) are shown in Figure 2. Fresh weights (FW) of the fruits sampled over the period were highest in the 0.2% OCD (18.9–21.7 g plant^−1^) and 0.4% OCD (18.8–21.3 g plant^−1^) treatments, significantly greater than those in control (15.7–20.1 g plant^−1^; *p* < 0.05; Figure 2A). For hardness, the greatest values were observed in the fruits sampled in 0.4% OCD (4.8–5.7 *N*) during the cultivation period, excluding February, which were significantly higher than those in control (4.0–4.5 *N*; *p* < 0.05; Figure 2B). In addition, the highest soluble solid content (SSC) values were found mostly in 0.2% OCD (9.3–12.0 °Brix) and 0.4% OCD (9.3–11.9 °Brix), significantly higher than those in control (7.9–10.9 °Brix; *p* < 0.05; Figure 2C). The most significant difference (approximately 1.23 fold) in the SSC values was found between 0.2% OCD and control treatments, particular in the final month. 

#### 2.2.2. Nutrients

Among the *Chlorella* application rates, 0.4% OCD treatment had the highest concentrations of P (6.97–8.04 g kg^−1^) in fruit samples over the five months (Table 2). Other *Chlorella* treatments induced intermediate concentrations of P (4.74–6.32 g kg^−1^), and control treatment had the lowest P concentration (3.51–5.09 g kg^−1^). Meanwhile, Ca concentration in the fruits showed the opposite trend; the highest value was observed mostly in control while 0.4% OCD had the lowest value. For other nutrients (Mg and K), it seemed that there was no significant difference in the concentrations among the treatments (*p* > 0.05) across the whole cultivation period.

#### 2.2.3. Sugars

**Figure 3:**
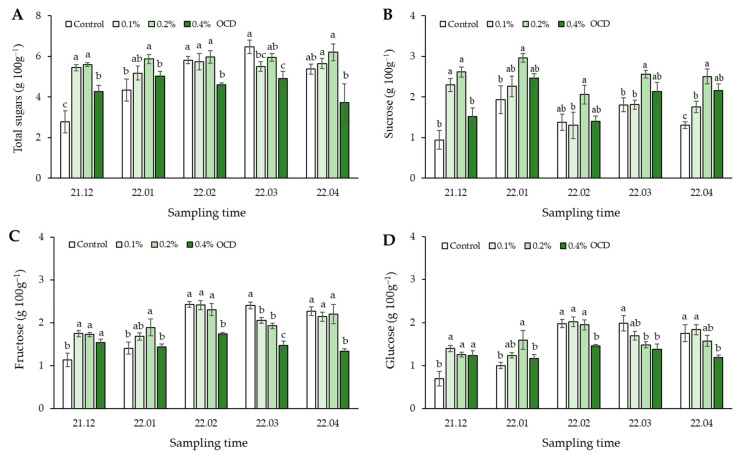
Monitoring sugar content and composition in strawberry fruits grown under different *Chlorella* application rates for five months: 0 (control), 0.1, 0.2, and 0.4% of the optimal cell density (OCD). Data are mean ± standard errors (*n* = 6). Different letters indicate significant difference among the treatments (Tukey’s HSD, *p* < 0.05). (**A**) Total sugars, (**B**) sucrose, (**C**) fructose, (**D**) glucose.

The highest contents of total sugars (TS) in strawberry fruits sampled in December 2021 and January 2022 were observed in 0.2% OCD (5.6–5.9 g 100 g^−1^), and were significantly higher than those in control (2.8–4.3 g 100 g^−1^) (*p* < 0.05; Figure 3A). On the other hand, in the remaining months, TS content in the fruits sampled in control represented the highest value (6.5 g 100 g^−1^) in March and the intermediate values (5.3–5.8 g 100 g^−1^) in February and April. Over the period, the lowest TS values were observed in 0.4% OCD (3.7–4.6 g 100 g^−1^).

The main types of sugar in strawberry fruits cultivated in control were fructose (41%), glucose (31%), and sucrose (27%). Meanwhile, the fruits harvested in 0.2% and 0.4% OCD treatments contained sucrose (43%), fructose (40%), and glucose (27%) in that order (Figure 3). Among these, sucrose contents were highest in 0.2% OCD across the whole cultivation period, significantly higher (34–66%) than those in control (*p* < 0.05; Figure 3B). Fructose and glucose contents were highest in control from February to April, and were significantly higher than those in 0.4% OCD (*p* < 0.05; Figure 3C,D).

#### 2.2.4. Organic Acids

**Figure 4:**
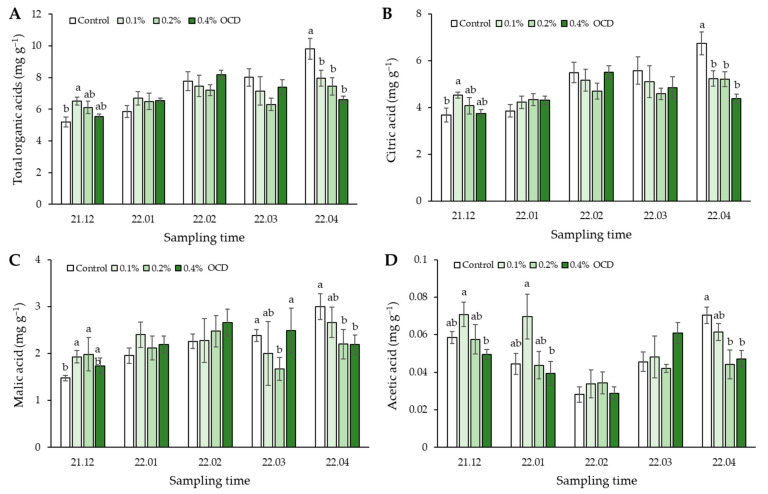
Monitoring organic acids content and composition in strawberry fruits grown under different *Chlorella* application rates for five months: 0 (control), 0.1, 0.2, and 0.4% of the optimal cell density (OCD). Data are mean ± standard errors (*n* = 6). Different letters indicate significant difference among the treatments (Tukey’s HSD, *p* < 0.05). (**A**) Total organic acids, (**B**) citric acid, (**C**) malic acid, (**D**) acetic acid.

The highest content of total organic acids (TOA) in strawberry fruits sampled in December 2021 was observed in 0.1% OCD (6.52 mg g^−1^) and was significantly higher than that in control (*p* < 0.05; Figure 4A). Over the next three months, TOA content in the fruits gradually increased in all *Chlorella* treatments, but there were no significant differences among the treatments (*p* > 0.05). TOA content in control then increased considerably (by approximately 23%) in April 2022 relative to the previous month, whereas this phenomenon did not occur in the remaining treatments. At this time, the TOA contents in the fruits were highest in control (9.8 mg g^−1^) and were significantly higher than those in the 0.1%, 0.2%, and 0.4% OCD treatments (6.6–8.0 mg g^−1^; *p* < 0.05).

Overall, the main organic acids in strawberry fruits were found to be citric acid (60–75%), malic acid (25–40%), and acetic acid (<1%). For citric acid, the highest value was observed in the 0.1% OCD treatment in December 2021 while the control treatment represented the highest value in April 2022 (Figure 4B). The remaining organic acids also showed a similar trend (Figure 4C,D). Consistently, these organic acids were found to be significantly lower (23–37%) in the 0.2% and 0.4% OCD treatments than in control (*p* < 0.05). Temporal changes in citric and malic acids appeared to be similar to TOA content, but not for acetic acid, which tended to drop highly in the third month of sampling.

#### 2.2.5. Fruit Flavor Quality

The highest sugar–organic acid (S:A) ratio of strawberry fruits was observed in 0.2% OCD (8.47–9.99) during the five month sampling period (Table 3). The lowest S:A ratio was found in control (5.26–7.49) in December, January, and April, while 0.4% OCD represented the lowest S:A ratio (5.77–6.83) in the remaining months. Likewise, the sweetness index (SI) of the fruits also showed a similar trend. The treatment of 0.2% OCD induced the highest SI values (8.76–10.0) in December, January, and April and these were significantly greater than those in control (4.59–8.75; *p* < 0.05).

#### 2.2.6. Correlations of Fruit Properties

**Figure 5:**
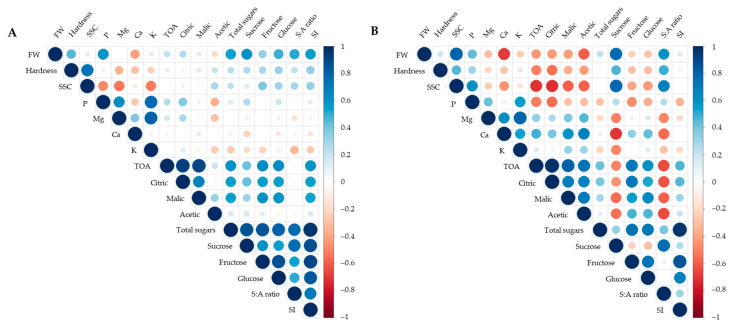
Correlation matrix of fruit properties sampled over the two months with the most favorable growing environment conditions during the monitoring period: (**A**) January 2022 and (**B**) April 2022. Positive and negative correlations are displayed in blue and red colors, respectively. Color intensity and the size of the circle are proportional to the Pearson correlation coefficients. The legend color on the right side of the correlogram indicates the correlation coefficient and the corresponding colors.

The correlations among the properties of fruits sampled in the second and fifth months, when there was the least damage from climate change and disease outbreaks during strawberry cultivation, are shown in Figure 5. At the early sampling (January 2022; Figure 5A), there were significant positive correlations of the fruits’ FW with hardness, P, TS, sucrose, and glucose (r = 0.45, 0.59, 0.54, 0.60, and 0.49, respectively, *p* < 0.05). In addition, SSC (°Brix) had a significant positive correlation with hardness (r = 0.73, *p* < 0.05) and negative correlations with P, Mg, and K (r = −0.49, −0.54, and −0.53, respectively, *p* < 0.05). Among sugar-related parameters, sucrose was significantly correlated to TOA, citric acid, TS, fructose, and glucose (r = 0.41, 0.42, 0.86, 0.60, and 0.54, respectively, *p* < 0.05). As an indicator of fruit flavor quality, the S:A ratio was significantly correlated to FW, TS, sucrose, fructose, and glucose (r = 0.51, 0.77, 0.76, 0.53, and 0.50, respectively, *p* < 0.05). 

On the other hand, the correlation trends among the variables of fruits sampled in April 2022 were different (Figure 5B). There was a significant positive correlation between fruit’s FW and SSC (r = 0.80, *p* < 0.001), but both variables had significant negative correlations with organic acids (r = −0.43 to −0.73, *p* < 0.05). In addition, the parameters related to sugars showed different correlations with organic acids: positive for fructose and glucose (r = 0.47 to 0.71, *p* < 0.05) and negative for sucrose (r = −0.46 to −0.56, *p* < 0.05). Sucrose was also negatively correlated with Mg, Ca, and K (r = −0.51, −0.73, and −0.50, respectively, *p* < 0.05). Moreover, the S:A ratio was positively correlated with FW, hardness, SSC, TS, and sucrose (r = 0.60, 0.49, 0.68, and 0.75, respectively, *p* < 0.01) but negatively correlated with organic acids (r = −0.60 to −0.66, *p* < 0.001). In contrast, the SI value showed a positive correlation with TOA and citric acid (r = 0.45, *p* < 0.05).

### 2.3. Anthocyanin Content

**Figure 6:**
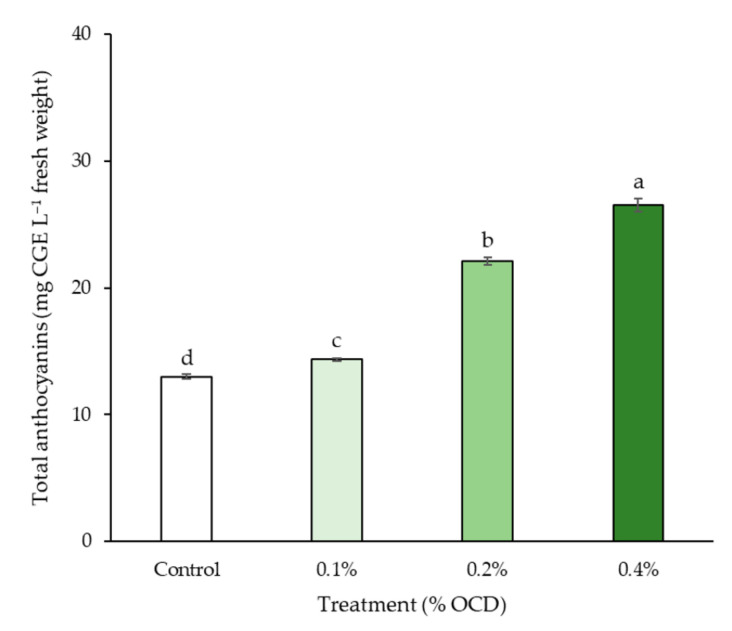
Anthocyanin contents in strawberry fruits sampled in different *Chlorella* application rates at the last sampling month: 0 (control), 0.1, 0.2, and 0.4% of the optimal cell density (OCD). Data are mean ± standard errors (*n* = 6). Different letters indicate significant difference among the treatments (Tukey’s HSD, *p* < 0.05).

Figure 6 shows the difference in anthocyanin content in strawberry fruits depending on *Chlorella* application rates. The highest anthocyanin content was observed in 0.4% OCD (27 mg 100 g^−1^), followed by the 0.2% OCD, 0.1% OCD, and control (13 mg 100 g^−1^) treatments. Compared with control, the anthocyanin contents in the fruits of the 0.2% and 0.4% OCD treatments were significantly higher (1.7 and 2.1 times, respectively) than that in control (*p* < 0.05). 

### 2.4. Postharvest Disease Incidence

**Figure 7:**
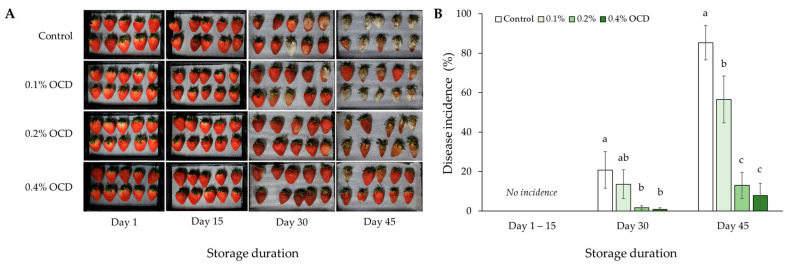
Incidence of grey mold disease (*Botrytis cinerea*) on strawberry fruits, sampled in different *Chlorella* application rates including 0 (control), 0.1, 0.2, and 0.4% of the optimal cell density (OCD), during 45 day storage test. (**A**) Temporal changes in the pathogen occurrence and severity on the fruits at 15 day intervals. (**B**) The incidence of grey mold disease is estimated as the proportion of pathogen-caused areas at 15 day intervals. Data are mean ± standard errors (*n* = 10). Different letters indicate significant difference among the treatments (Tukey’s HSD, *p* < 0.05).

As a result of the postharvest fruit storage test, no occurrence of grey disease incidence caused by *Botrytis cinerea* was found in strawberries during the first two weeks (Figure 7). Twenty-five days after the storage, the pathogen was first observed on the surface of fruits in the control and 0.1% OCD treatments, and the disease incidence in both treatments increased sustainably until the last day (Day 45) of the storage test. Over the storage duration, the final incidences of grey disease according to the *Chlorella* application rates were listed in the following order: control (85%), 0.1% OCD (57%), 0.2% OCD (13%), and 0.4% OCD (8%) treatments. 

### 2.5. Principal Component Analysis

**Figure 8:**
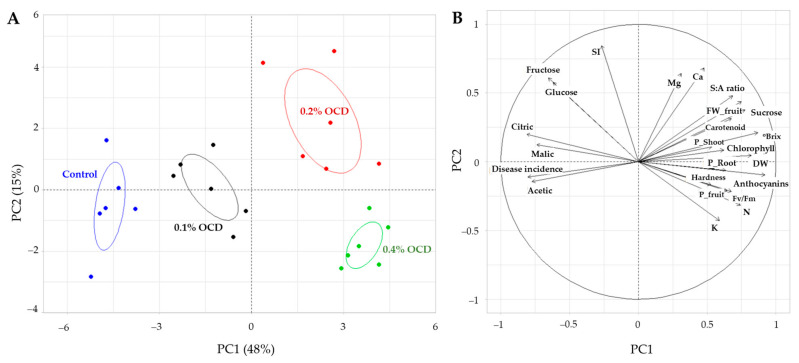
Principal component analysis (PCA) of plant and fruit properties in treatments of different *Chlorella* application rates: 0 (control), 0.1, 0.2, and 0.4% of the optimal cell density (OCD). (**A**) Scores for the first two principal components of four different application rates. (**B**) Loading plot of the parameters related to plant physiology and fruit quality.

A clear separation of *Chlorella* treatments was confirmed through the principal component analysis (PCA) result of the dataset for plant and fruit properties (Figure 8). Along the PC1 axis (48% variance), the treatments with different OCD levels were separated primarily due to variables of plant physiology (e.g., DW, chlorophyll, carotenoid, and Fv/Fm) and nutrients (e.g., N and P) as well as fruit quality, such as SSC, FW, hardness, components (e.g., sucrose, organic acids, S:A ratio, and anthocyanins), and disease incidence (*p* < 0.01). Along the PC2 axis (15% variance), the treatments were split by the sweetness index, Mg and Ca in the plants, and fructose and glucose in the fruits (*p* < 0.01). The overall trend of this multivariate analysis indicates that *Chlorella* application at the level of 0.2% OCD induced the most positive impacts on the major variables of plant growth and fruit quality, as well as their significant correlations.

## 3. Discussion

In modern agriculture, microalgae have been widely used as biofertilizers, biostimulants, and biopesticides because they contain essential sources of nutrients (e.g., N, P, K, etc.), primary and secondary metabolites (e.g., polysaccharides, phenolics, phytohormones, vitamins, etc.), and bioactive compounds to various environmental conditions (e.g., antimicrobial, antioxidant, antiviral or antifungal substances) [31], thereby improving soil fertility and quality and crop yield and performance. As such, in the present study, the improvements in growth performance and nutrient uptake efficiency of strawberry plants by *Chlorella* application were confirmed through increased biomass and nutrient contents (Figure 1) and stimulated photosynthetic activity and pigmentation (Table 1). According to our previous analysis of *C. fusca* CHK0059 cell composition, it contained high levels of N (91.2 g kg^−1^), P (9.38 g kg^−1^), K (2.62 g kg^−1^), Ca (1.05 g kg^−1^), Mg (5.85 mg kg^−1^), etc., inferring that added *Chlorella* is likely to be well absorbed as a nutrient source for strawberry plants. In addition, the improved nutritional status of the plants appeared to positively influence the photosynthetic capacity by regulating their morphological characteristics, such as specific leaf area and leaf mass ratio, that are highly related to the efficiencies of light capture and carbon gain [32,33]. A similar research result has also been reported by Kim et al. [26], who demonstrated significant increases in shoot height, leaf thickness, and leaf pigments of the strawberry cultivar ‘Seolhyang’ by *C. fusca* CHK0059 application. Moreover, the promoting effect on plant growth performance by microalgae application has been reported in various crops, such as wheat, rice, maize, lettuce, tomato, cabbage, etc., as reviewed in Lee and Ryu [23] and Çakirsoy et al. [34]. 

Strawberry is one of the most popular fruits in the world because of its sweetness, fragrant flavor, juicy texture, and health-promoting compounds and fibers [35], so it is currently considered a high-value fruit crop that is important for agriculture and local economies, especially in South Korea. With the high demand for better-tasting, longer-lasting, and healthier fruits from consumers, various agricultural technologies including *Chlorella* fertilization, have been applied to strawberry cultivation. In this respect, the application of *C. fusca* CHK0059 (0.2% and 0.4% OCD) in the present study led to considerable increases in the SSC (°Brix), hardness, and biomass of strawberry fruits throughout the entire cultivation (Figure 2), suggesting its superior role in the improvement of fruit quality and yield. Additionally, positive correlations of fruit SSC and hardness with P concentrations in roots, shoots, and fruits may (Figure 8) indicate that highly absorbed P in plant shoots and edible parts following *Chlorella* application also affected the fruit quality parameters. In general, phosphorus is an important macronutrient for photosynthesis and affects sugar partition and transportation as a form of phosphate (Pi) [36]. In this context, the improvement of P status in strawberry roots and shoots following *Chlorella* application seems to significantly stimulate the overall photosynthetic functions (see Table 1), contributing to an increase in sugar accumulation in fruits with high P.

In general, fruit flavor is determined by the quantity and type of sugars, organic acids, and volatile compounds. A good balance of these substances will meet the consumer preference; however, among these, sugars corresponding to the sweetness intensity of fruits are the most significant determinant. In ripe strawberries, the predominant soluble sugars are sucrose, fructose, and glucose, and the content and composition of these have a decisive effect on determining fruit quality [37] but vary with genotypes, development stages, farming practices, and environmental conditions [38]. In this study, the sugar type that responded most positively to *Chlorella* application during strawberry cultivation was sucrose (Figure 3). Sucrose is known to be the ultimate product of photosynthesis. This carbohydrate is converted from all photo-assimilates that are not required for leaf function and transported into sink organs, i.e., roots, shoots, and fruits, through the phloem, leading to coordinating physiological growth and development [39]. The amount of sucrose that can be released from the sources to the receptacle fruit sink via long-distance transport depends on various factors: e.g., photosynthetic activity, partitioning between starch synthesis within the chloroplast and triose-Pi moved out of the chloroplast for sucrose synthesis, and temporary storage of sucrose in the vacuole [37,40,41]. In our findings, a significant correlation of sucrose in fruits with Fv/Fm, chlorophylls, carotenoids, and P in leaves (Figure 8) suggests that the application of *C. fusca* CHK0059 could act as a promoter for fruit flavor as well as for plant growth by increasing photosynthetic efficiency and Pi availability, which are highly related to the sucrose synthesis. Additionally, increased N, Mg, and K contents in the strawberry plants treated with *C. fusca* (Figure 1) seem to promote overall metabolic processes, including photosynthesis, pigmentation, and carbohydrate accumulation in the leaves, subsequently facilitating the sucrose generation and transport into the sink organ [41]. On the other hand, fructose and glucose contents in the fruits showed similar responses to technological (i.e., *Chlorella* application rate) and temporal changes, but these were negatively correlated to sucrose content in the fruits (Figure 5 and Figure 8). A similar result has also been observed by Schwieterman et al. [38]. In addition, the S:A ratio as a taste index was positively correlated with sucrose but not with other sugars in the fruits (Figure 5B), suggesting that the increased sucrose content following *Chlorella* application led to an improvement in the overall strawberry flavor intensity. Furthermore, Schwieterman et al. [38] have reported a high correlation between sucrose and total volatiles, relevant to fruit aroma, in 35 strawberry cultivars. In light of this, in the present study we expect that *Chlorella* application probably also led to an improvement in total volatile substances, which will increase consumer choice. 

Besides sweetness, sourness is also a crucial factor in determining strawberry fruit flavor associated with organic acids [30]. In general, organic acids can mediate various functions in plant growth and development by supporting photosynthesis and stomata regulation as well as acting as an alternative energy source, in turn, the metabolic processes contribute to fruit development and quality [42,43]. Additionally, they also play an important role in the gelling properties of pectin in fruit [44]. In the present study, we found an increase in TOA contents, mainly citrate and malate, in the strawberry fruits treated with *C. fusca* CHK0059 (Figure 4), inferring that *Chlorella*-induced organic acids induced benefits not only for plant development but also for development, maturation, and ripening of strawberries [42]; however, such an effect was represented during the initial two or three months but not the remaining period. The qualitative and quantitative variations of organic acids are usually dependent on cultivars, genotypes, and maturation stages of strawberry fruits [45]. Additionally, the difference in accumulation of these organic acids in the fruits may also be attributed to environmental fluctuations, including temperature, ambient CO_2_, and water and mineral supplies, during the cultivation experiment [46]. Despite the biochemical variation in strawberry fruits, and considering that 0.2% OCD of *C. fusca* CHK0059 led to the highest values of two indices, i.e., S:A ratio and SI, during the entire cultivation period (Table 3), it seems that the effect of *Chlorella*-induced sugar production on the fruit flavor quality far outweighed the effect on organic acid production in this study.

It is known well that anthocyanins are responsible for the red color in strawberries and also serve as health-promoting substances with antioxidant, anticancer, and anti-inflammatory properties [47], thus directly affecting fruit appearance and consumer choice. In the present study, like sucrose, the most noticeably improved fruit parameter with the application of *C. fusca* CHK0059 was anthocyanins, which tended to increase with increasing application rate (Figure 6). Similarly, Roussos et al. [48] have reported that the application of brown alga (*Ascophyllum nodosum*) extract induced a higher content of anthocyanins in strawberry cv. Camarosa. As is widely known, the accumulation of anthocyanins in fruits is affected by sucrose because it not only provides the energy source for the synthesis of anthocyanins but also acts as a signaling molecule inducing transcription of key enzymes (e.g., G6PHD, SKDH, PAL, C4H, 4CL, etc.) related to the biosynthesis pathway [49,50]. In addition to that evidence, the stimulatory effects of sucrose on the biosynthesis of anthocyanins have been documented in various plant species, including strawberries [49,50], grapes [51], broccoli sprouts [52], etc. Likewise, in this study, we found a high correlation between anthocyanins and sucrose contents in the strawberry fruits (Figure 8). 

Typically, strawberries consisting of soft tissue are highly susceptible to the attacks of pathogens, including viruses, bacteria, fungi, and nematodes, especially during postharvest storage [47,53]. Among pathogens, the ascomycete *B. cinerea* is regarded as the primary disease of strawberries, causing a significant economic loss in the global strawberry industry. In contrast with such fruit disease damage, the application of *C. fusca* CHK0059 used in this study induced a marked antifungal effect on the postharvest strawberry fruits (Figure 7). Considering the PCA result that showed a significant negative correlation between anthocyanins and disease incidence in fruits (Figure 8), increased anthocyanin accumulation in fruits with the application of *C. fusca* CHK0059 appeared to have acted as an antifungal factor that effectively suppressed the occurrence of grey mold disease during the postharvest storage test. Likewise, reduced susceptibility to postharvest fungal pathogens, including *B. cinerea,* has been reported in other fruits enriched in anthocyanins: grapes [54], mango [55], and tomato [56]. In plants, anthocyanins are produced with other classes of flavonoids, e.g., flavones, flavonols, and tannins, via the same biochemical pathway [57]. Among these defensive secondary compounds, tannins can function to protect plants from both abiotic (e.g., cold, light, etc.) and biotic (e.g., pathogens and herbivores) stresses, and they share the same precursors, i.e., leucoanthocyanins, in the biosynthesis pathway with anthocyanins [54,57,58]. Therefore, it can be assumed in this study that the *Chlorella* application resulted in a simultaneous increase in these two metabolites in strawberry fruits, consequently enhancing the defensive capacity of the postharvest fruits to pathogen attacks. In addition to pathogen resistance, anthocyanin-induced tolerance of postharvest fruits to oxidative stress generally caused by wound and senescence may delay the pathogen infection and development [56].

## 4. Materials and Methods

### 4.1. Chlorella Inoculation

For *Chlorella* inoculation, *Chlorella fusca* CHK0059 was provided by the Organic Agriculture division of the National Institute of Agricultural Sciences, RDA in South Korea. The *Chlorella* cells were placed into commercial mineral water (Ca—25 mg L^−1^; Na—5.3 mg L^−1^; K—1.6 mg L^−1^; Mg—3.0 mg L^−1^) and cultured at the level of optimal cell density (1.0 × 10^7^ cells mL^−1^) recommended by the RDA [24]. The *Chlorella* inoculation was conducted in a greenhouse of the experimental farm during the strawberry cultivation, and the culturing conditions were as follows: temperature (25 ± 3 °C), natural sunlight-dependent, and continuous air supply. 

### 4.2. Plant Cultivation and Chlorella Treatment

A total of 800 seedlings of strawberry (*Fragaria ananassa* Dutch. cv. ‘Kuemsil’) were planted in raised beds (ca. 50 cm width) containing artificial soil substrate and cultivated from October 2021 to April 2022 in a greenhouse located in Jinju, South Korea (35°13′35″ N, 127°57′32″ E). The planting distance between the seedings was about 15 cm, and they were planted in two rows on the beds. The nutrient solution was supplied four times daily via an irrigation system and its composition was formulated based on the RDA method [59]. The pH of irrigated water was 5.8–6.0, and the electrical conductivity was adjusted according to the growth stage of strawberry plants: 0.8–1.5 dS m^−1^ (vegetative to budding) and 1.2–1.8 dS m^−1^ (flowering to ripening). During the cultivation period, the temperature in the greenhouse was controlled to maintain the following levels: 26 °C for daytime and more than 8 °C for nighttime. Additionally, the management of flower number was conducted as 5–7 flower buds per inflorescence, and misshapen fruits also were cut off. 

There were four different application rates of *C. fusca* CHK0059 culture solution to strawberry seedlings: 0% (control), 0.1%, 0.2%, and 0.4% of OCD. The *Chlorella* solutions were applied weekly to the soil substrate through an irrigation system. The appropriate level of *Chlorella* solution recommended by the RDA for strawberry cultivation is 0.2% OCD [60], so the application rates for this study were installed based on this. 

### 4.3. Fruit and Plant Collection

Strawberry fruits, with 80–90% of the fruit surface showing a visible red color [59], were sampled weekly for five months, from December 2021 to April 2022. Of those, up to 50 individuals weighing 15 ± 5 g were selected per treatment each month and their FW recorded. Then, analysis for other fruit characteristics was performed using six fruit samples: e.g., hardness, SSC, total sugars and components (sucrose, fructose, and glucose), total organic acids and components (citric acid, malic acid, and acetic acid), nutrients (P, Mg, Ca, and K), and anthocyanins. 

Plant sampling was performed after the last fruit harvest in April 2022. Six individuals of each *Chlorella* treatment were selected, and their photosynthesis rates were measured in situ. Then, the plant samples were collected and transferred to a laboratory of Gyeongsang National University to determine other plant characteristics, such as morphology, dry weight, photosynthetic pigments (Chl a, Chl b, total Chl, and carotenoid), and nutrients (N, P, Mg, Ca, and K). 

### 4.4. Grey Mold Incidence during Postharvest Storage

Strawberry fruits harvested in the last sampling month were stored at 4 °C and 80–90% relative humidity in a cold chamber (JSSC-700C, JS Research Inc., Gongju, Republic of Korea). Afterward, the occurrence of grey mold disease (*Botrytis cinerea*) on the fruit surface was observed for 45 days. Each *Chlorella* treatment had 10 individual fruits. The disease incidence (%) of strawberry fruits was estimated as a proportion of pathogen-caused area to total area, the scale of which was measured using the ImageJ v. 1.51i software (https://imagej.nih.gov/ij/index.html: accessed on 1 March 2023). The postharvest storage assay was conducted in duplicate.

### 4.5. Analytical

The hardness (*N*) and SSC (°Brix) of collected fruits were recorded using a fruit hardness tester (KM-1, Fujiwara Factory, Tokyo, Japan) and a Brix refractometer (Surakan SRS03, Snix Electronics Co., Ltd., Cheongju, Republic of Korea), respectively. 

The photosynthesis rate in the leaves of strawberry plants was measured using a portable fluorometer with leaf-clip holders (MINI–PAM–II, Heinz Walz GmbH, Effeltrich, Germany). In addition, the photosynthetic pigments, such as chlorophylls and carotenoids, in the leaves were determined following the extraction method with 80% acetone [61]. The extract was measured at 470, 645, and 663 nm wavelengths with a UV spectrophotometer (UV–160A, Shimadzu, Kyoto, Japan) and the pigments of Chl a, Chl b, total Chl (a + b), and carotenoids were estimated with the following formula [1]:

Chl a = 12.7 A663 − 2.69 A645

Chl b = 22.9 A645 − 4.68 A663

Total Chl (a + b) = 20.29 A645 + 8.02 A663

Carotenoids = (1000 A480 − 1.82 Chl a − 85.02 Chl b)/198

where A is the absorbance value at the appropriate wavelength.

The N concentration in plant samples was determined by a Kjeldahl distiller (K–355, BÜCHI, Flawil, Switzerland) following wet digestion with H_2_SO_4_–HClO_4_ solution [62]. To determine the concentrations of nutrients, including P, K, Ca, and Mg, in plants and fruits, the samples were digested with HNO_3_–H_2_O_2_ solution and then determined using ICP–OES (iCAP Pro XP Duo, Thermo Scientific, Waltham, MA, USA) [62].

For carbohydrate analyses, dried fruit samples (100 mg) were extracted with 10 mL of distilled water heated for four hours in a water bath at 60 °C and then centrifuged for 15 min at 3000 rpm. The supernatant was filtered with a 0.45-µm syringe filter and the contents of organic acids, such as citric, malic, and acetic acids, were determined using ultra-high performance liquid chromatography (UHPLC) (Dionex Ultimate 3000, Thermo Fisher Scientific Inc., Dreieich, Germany) by injection into a SunFire TM C18 column (4.6 mm × 250 mm, 5 µm, Waters, Milford, MA, USA). The mobile phase used was 0.02 N H_2_SO_4_ and the flow rate was 1 mL min^−1^. The soluble organic acids were detected with a variable wavelength detector (VWD-3100, Thermo Fisher, Crawley, UK). The sum of these three organic acids was considered as TOA. 

The supernatant extracted from the fruit samples above was also used to determine soluble sugars according to a method of Keutgen and Pawelzik [30]. Contents of fructose, glucose, and sucrose in the extracts were determined using an Agilent 1200 series HPLC (Agilent, Santa Clara, CA, USA) equipped with a refractive index detector (RID) following injection of the fruit extracts (20 µL) into an Agilent Zorbax carbohydrate column at 30 °C (4.6 mm × 150 mm, I.d., 5 µm). The mobile phase used was 75% acetonitrile and the flow rate was 1.4 mL min^−1^. The sum of fructose, glucose, and sucrose was considered TS. 

Anthocyanin content in strawberry fruit was determined with the pH differential method [63]. The frozen sample was mixed thoroughly with 0.025 M potassium chloride buffer (pH 1.0) and 0.4 M sodium acetate buffer (pH 4.5) and then incubated for 90 min at room temperature. After centrifugation, the absorbance of the supernatant was measured at 520 and 700 nm wavelengths with a UV spectrophotometer (UV–160A, Shimadzu, Japan). The following formula was used to calculate the anthocyanin content expressed as cyanidin-3-glucoside equivalents (CGE):

$$\mathrm{Anthocyanin}\;\left({\mathrm{mg}\;\mathrm{CGE}\;L}^{-1}\;\mathrm{FW}\right)=\frac{A\;\times \;\mathrm{MW}\;\times \mathrm{DF}\;\times 10^{3}}{\varepsilon \;\times \;1}$$

where A: (*A*_520nm_ − *A*_700nm_)_pH1.0_ − (*A*_520nm_ − *A*_700nm_)_pH4.5_; MW (molecular weight, g mol^−1^) = 449.2; DF = dilution factor; 10^3^ = conversion factor from g to mg; ε = 26,900 (molar extinction coefficient for cyanidin-3-glucoside, L^−1^ mol^−1^ cm^−1^); 1 = pathlength (cm).

### 4.6. Fruit Quality Index

To assess the quality of fruit flavor, i.e., sweetness, data for sugar and organic acids in strawberries were used. To characterize it, the S:A ratio [64] was calculated with the following formula: TS content (sucrose + fructose + glucose)/TOA content (citric + malic + acetic acids). In addition, the sweetness of strawberries was evaluated by a method of Keutgen and Pawelzik [30]. The sweetness index of fruits was calculated based on the degree to which each carbohydrate contributes to overall sweetness as follows: SI = (1.0 × glucose) + (2.3 × fructose) + (1.35 × sucrose). 

### 4.7. Statistical Analysis

To compare differences in variables of plant and fruit characteristics among different *Chlorella* treatments, one-way ANOVA with Tukey’s honestly significant difference (HSD) post hoc test at the 0.05 probability level was conducted (*n* = 50 for FW and 6 for others). Pearson correlation analysis was conducted to investigate the correlations among the properties of fruits sampled in the second and last sampling months. Principal component analysis was performed to investigate patterns of variation in the dataset, focusing on the degree of relationships among key parameters related to plant performance and fruit quality throughout different *Chlorella*-level treatments. All statistical analyses in the present study were performed using the R program (version 3.3.3).

## 5. Conclusions

Microalgae are known to be of great utility for agricultural crop production, but their efficiency may vary depending on the species and treatment levels. Our findings obtained from the current fieldwork demonstrate that *C. fusca* CHK0059 has a high value as a biofertilizer resource for strawberry cultivation. Application of *C. fusca* CHK0059 stimulated the nutrient uptake efficiency (e.g., N, P, K, Mg, etc.) and physiological characteristics (e.g., photosynthesis, pigments, and biomass) of strawberry plants, leading to improvements in the fruit characteristics (e.g., SSC and hardness) and flavor quality (e.g., S:A ratio and SI). Among the fruit-related parameters, sucrose and anthocyanin contents tended to show the greatest response to *Chlorella* application, indicating that these substances may be key factors in improving the quality of strawberry fruits during the whole cultivation period. Additionally, the elevated content of anthocyanins in fruits following *Chlorella* application appeared to serve as antifungal agents for postharvest strawberries. Overall, the application of *C. fusca* CHK0059 could promote the overall growth and performance of strawberry plants, contributing to the improvement of strawberry quality and yield as well as postharvest storage capacity, especially in 0.2% OCD treatment. Further studies are needed to specifically identify the mechanisms of the effects of *Chlorella* fertilization through analysis of the interactions among various factors that determine the quality of strawberry fruits. 

## Figures and Tables

**Table 1:** The maximum photosystem II quantum yield (Fv/Fm) and photosynthetic pigments in leaves of strawberry plants grown under different *Chlorella* application rates.

Treatment (% OCD)	Fv/Fm	Chlorophyll a (mg 100 g^−1^)	Chlorophyll b (mg 100 g^−1^)	Total Chlorophyll (mg 100 g^−1^)	Carotenoids (mg 100 g^−1^)
Control	0.813 b	0.580 b	0.195 b	0.777 b	1.923 b
0.1%	0.813 b	0.628 ab	0.205 ab	0.843 ab	2.257 a
0.2%	0.834 a	0.656 ab	0.208 ab	0.865 ab	2.406 a
0.4%	0.840 a	0.683 a	0.235 a	0.920 a	2.338 a

Data represent the mean values of six replicates. Different letters indicate significant difference among the treatments (Tukey’s HSD, *p* < 0.05). OCD, the optimal cell density of *Chlorella fusca* CHK0059.

**Table 2:** Concentration of nutrients, including P, Mg, Ca, and K, in strawberry fruits grown under different *Chlorella* application rates for five months.

Element	Treatment (% OCD)	Sampling Time				
		21.12	22.01	22.02	22.03	22.04
P (g kg^−1^)	Control	3.61 c	3.81 c	3.51 c	3.70 c	5.09 b
	0.1%	5.62 b	5.57 b	5.39 b	4.99 b	6.14 ab
	0.2%	6.25 ab	6.03 b	4.74 bc	5.69 b	6.32 ab
	0.4%	6.97 a	8.04 a	7.44 a	7.15 a	7.64 a
Mg (g kg^−1^)	Control	1.62 a	1.45 a	1.51 ab	1.62 a	1.81 a
	0.1%	1.63 a	1.50 a	1.55 ab	1.57 a	1.69 a
	0.2%	1.49 a	1.41 a	1.34 b	1.47 a	1.56 a
	0.4%	1.37 a	1.53 a	1.65 a	1.58 a	1.71 a
Ca (g kg^−1^)	Control	3.29 a	3.70 a	2.99 a	3.26 a	2.61 a
	0.1%	3.19 a	2.54 ab	2.57 a	3.14 ab	2.75 a
	0.2%	2.59 ab	2.67 b	2.52 a	2.35 ab	1.89 b
	0.4%	1.85 b	2.07 b	2.18 a	2.26 b	2.01 b
K (g kg^−1^)	Control	15.4 a	12.4 b	11.1 b	12.3 b	17.0 a
	0.1%	14.8 a	13.9 ab	13.5 ab	13.2 ab	16.1 a
	0.2%	14.2 a	12.3 b	11.4 b	13.4 ab	15.0 a
	0.4%	14.1 a	15.2 a	16.6 a	15.3 a	16.7 a

Data represent the mean values of six replicates. Different letters indicate significant difference in the concentration of each element among the treatments at each sampling time (Tukey’s HSD, *p* < 0.05). OCD, the optimal cell density of *Chlorella fusca* CHK0059.

**Table 3:** Assessment of fruit flavor quality of strawberries grown under different *Chlorella* application rates for five months.

Quality Index	Treatment (% OCD)	Sampling Time				
		21.12	22.01	22.02	22.03	22.04
S:A ratio	Control	5.26 b	7.49 b	7.61 ab	7.92 ab	5.61 c
	0.1%	8.38 a	7.76 b	7.96 a	8.39 ab	7.30 b
	0.2%	9.36 a	9.99 a	8.81 a	9.58 a	8.47 a
	0.4%	7.77 a	7.76 b	5.77 b	6.83 b	7.12 b
SI	Control	4.59 c	6.85 b	9.41 a	9.97 a	8.75 b
	0.1%	8.53 a	8.16 ab	9.33 a	8.90 b	9.14 ab
	0.2%	8.76 a	9.95 a	10.0 a	9.37 ab	10.0 a
	0.4%	6.83 b	7.81 b	7.38 b	7.64 c	7.18 c

Data represent the mean values of six replicates. The fruit quality was evaluated by determining the sugar–organic acid (S:A) ratio and the sweetness index (SI) based on the method of Keutgen and Pawelzik [30]. Different letters indicate significant difference among the treatments (Tukey’s HSD, *p* < 0.05). OCD, the optimal cell density of *Chlorella fusca* CHK0059; S:A, total sugars to total organic acids; SI, the sweetness index.

## Data Availability

Data are contained within the article.
